# Supplementary material for: A Novel LHX6 Reporter Cell Line for Tracking Human iPSC-Derived Cortical Interneurons
Source: Cells. 2022 Mar 1;11(5):853. doi: 10.3390/cells11050853 (PMC8909769; doi:10.3390/cells11050853)
Supplement: Supplementary file 1 [file cells-11-00853-s001.zip › cells-1537704-supplementary.pdf]

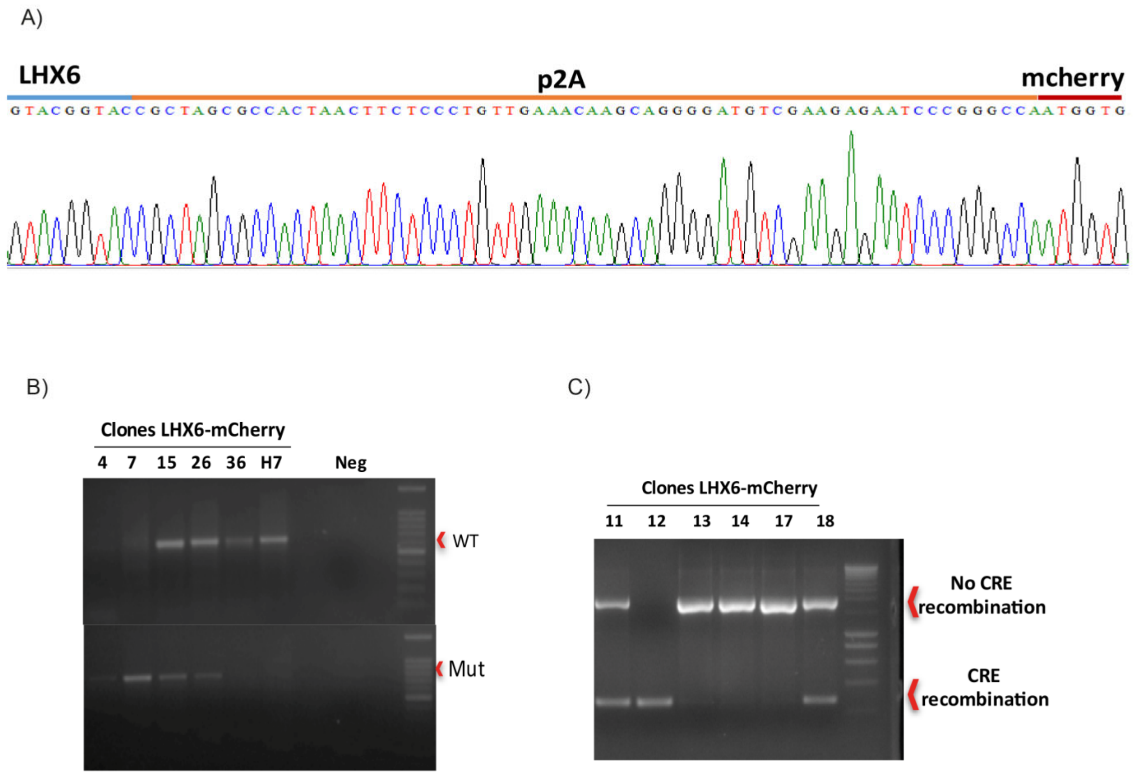

Figure S1. Genotyping of the LHX6-mCherry lines. **A**, An example of sequence read of 5' PCR product in a targeted clone confirming in frame integration of p2A-mCherry immediately downstream of the last coding codon of LHX6. **B**, Genomic PCR detection of the WT and targeted allele using the 5' primer pairs. **C**, PCR verification of Neo cassette removal.

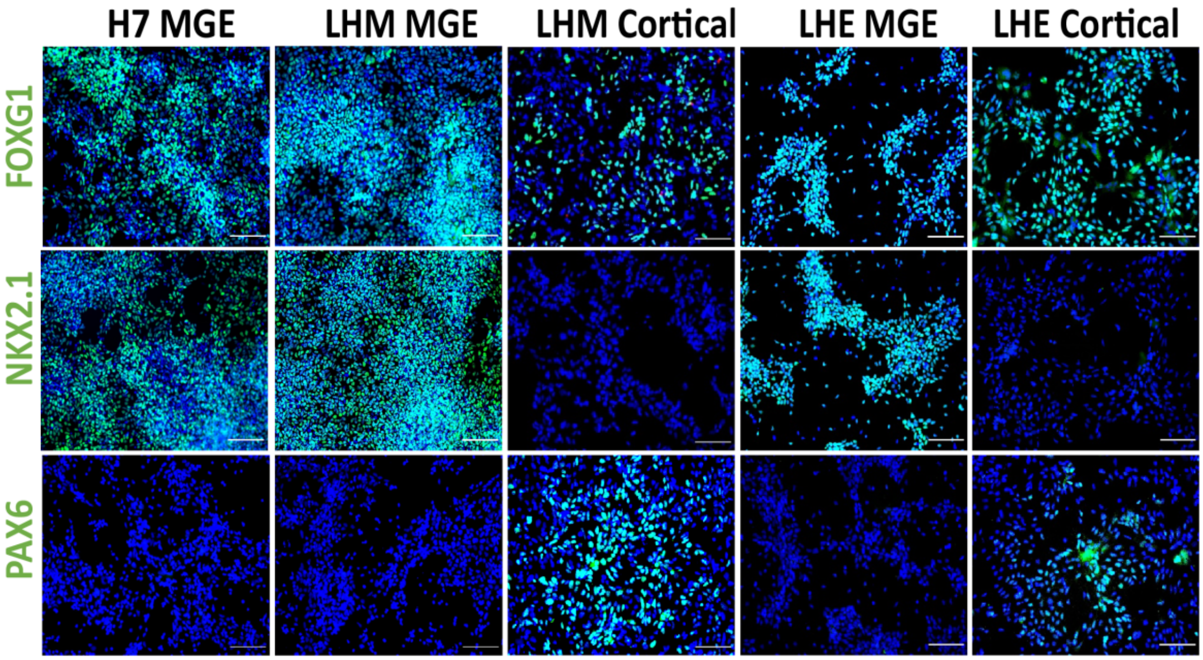

Figure S2. Additional information for figure 3. A larger canvas of figure 3B

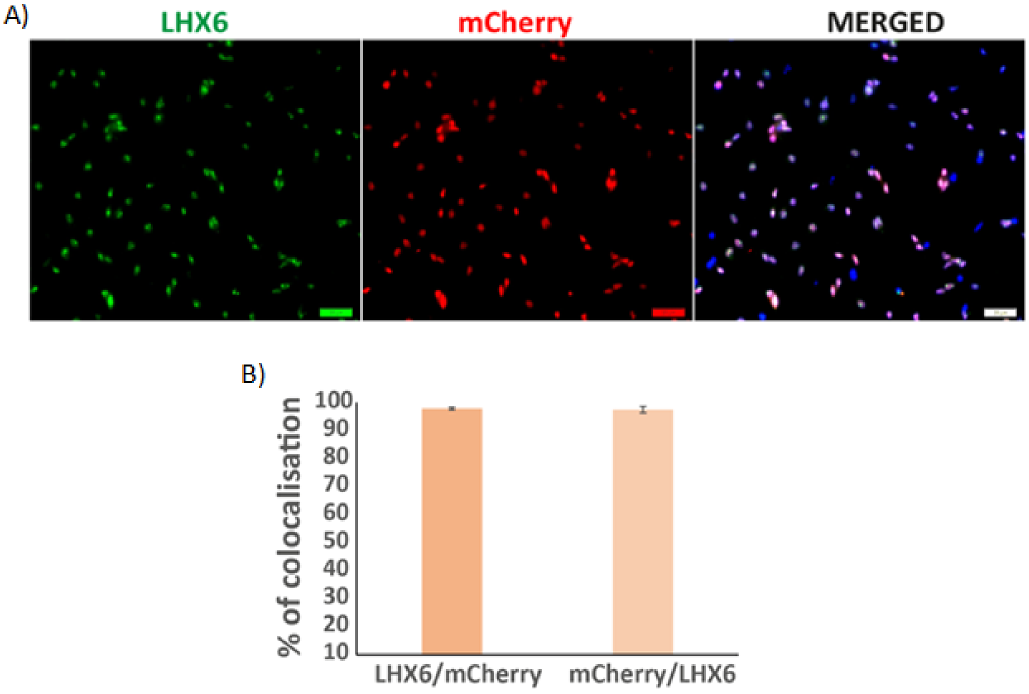

Figure S3. Additional information for figure 3E. **A**, Color-by-color images of Figure 3E.**B**, quantification of LHX6 and mCherry coexpression.

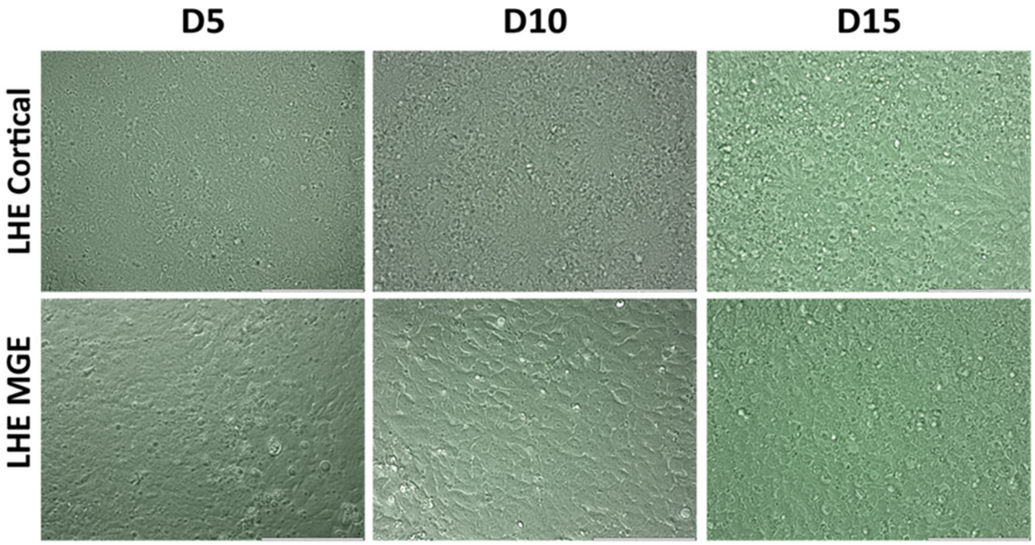

Figure S4. Expression of LHX6-mEmerald reporter during MGE differentiation. No detectable mEmerald fluorescence signal in day 5-15 MGE differentiation cultures

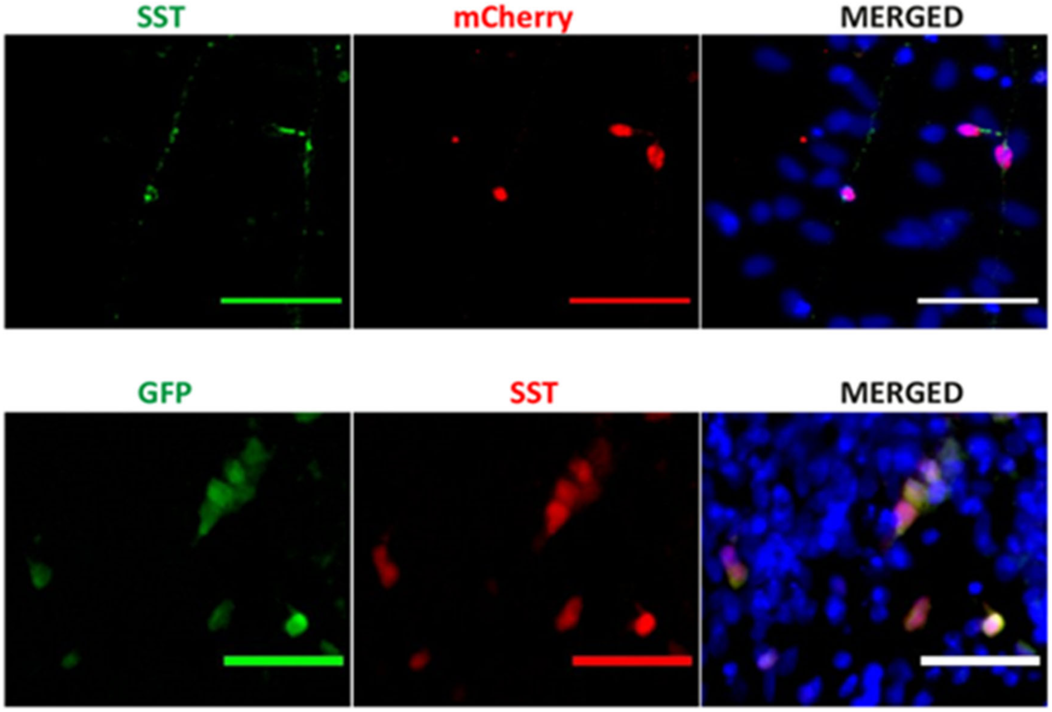

Figure S5. Additional information for figure 3. Color-by-color images of figure 3H and 3I.

Tables 1. Summary of CNV analysis

Cell lines	Copy number variance detected (>100kb and spanning >10 SNPs)
**KOLF2**	3p14.2dup, 3p13del, 6p22.3del, 9q33.1del, 18q22.1dup
**LHE1**	3p14.2dup, 3p13del, 6p22.3del, 9133.1del, 18q22.1dup
**LHE2**	3p14.2dup, 3p13del, 6p22.3del, 9133.1del, 18q22.1dup
**H7**	2q37.3del, 3q26.1dup, 2q37.3del
**LHM1**	2q37.3del, 3q26.1dup, 2q37.3del, 17p13.1del
**LHM2**	2q37.3del, 3q26.1dup, 2q37.3del, 17p13.1del, 20q11.21dup

DNA samples of LHE and LHM lines and their respective isogenic control lines were genotyped on Illumina Global Screening Array v3.0 and data analysed using PennCNV with GRCh37/hg19 as a reference genome. Note that no CNVs were detected in the *LHX6* containing region 9q33.2. Del=deletion, Dup=duplication.
